# A mini review on selenium quantum dots: synthesis and biomedical applications

**DOI:** 10.3389/fbioe.2023.1332993

**Published:** 2023-12-21

**Authors:** Yanhua Huang, Guangming Lu, Li Zhou

**Affiliations:** Guangxi Key Laboratory of Optical and Electronic Materials and Devices, Guangxi Colleges and Universities Key Laboratory of Natural and Biomedical Polymer Materials, and College of Materials Science and Engineering, Guilin University of Technology, Guilin, China

**Keywords:** selenium quantum dots, fluorescence, bioimaging, biosensing, diagnosis

## Abstract

In recent years, the demand for advanced biomedical nanomaterials has seen a noticeable surge. Among the essential trace elements in the human body, selenium has gained recognition for its anti-cancer, antioxidant, and immune regulatory properties. However, traditional selenium-based semiconductor quantum dots (QDs) are often comprised of heavy metal elements that tend to be toxic, thereby limiting their usage in biomedical applications. Fortunately, the advent of elemental selenium quantum dots (SeQDs), a new kind of fluorescent nanomaterial with unique physicochemical properties, has provided a solution to this problem. These SeQDs are known for their low toxicity and good biocompatibility, making them a promising candidate for biomedical applications. In this mini-review, we delve into the synthesis methods of fluorescent SeQDs and the latest progress in their applications in bioimaging, biosensing, and diagnosis treatment. Finally, we identify the major challenges and future prospects in the field of SeQDs.

## Introduction

Recent years have witnessed a surge of interest in fluorescent quantum dots (QDs), which are tiny structures made of semiconductor nanocrystals. The QDs offer striking advantages, such as remarkable optical, electrical, electrochemical, and catalytic properties, thus proving to be highly versatile for an array of practical applications (Lin et al., 2022; Triana et al., 2022). However, the conventional QDs typically contain heavy metals from the II-IV or III-V groups of the periodic table, making them unsuitable for applications in biological and environmental fields due to their toxic nature. Consequently, there is an urgent need to develop alternative QDs that are composed of non-toxic materials and that can be used in a variety of new application areas (Yao et al., 2018; Gao et al., 2020; Ruan and Zhou, 2022). As such, the development of metal-free fluorescent QDs, such as carbon QDs (Zhou et al., 2013; Liao et al., 2016; Lu et al., 2018), graphene QDs (Zhou et al., 2017), sulfur QDs (Song et al., 2019; Wang et al., 2019; Arshad and Sk, 2020; Gao et al., 2022), and silicon QDs (Yang et al., 2023) has garnered significant interest as they present viable alternatives to traditional semiconductor QDs, with the added benefit of being non-toxic and able to be applied in diverse fields.

Selenium (Se) is considered to be one of the essential trace elements for the human body (Huang et al., 2020b). However, the practical applications of semiconductor quantum dots containing selenium elements, such as cadmium selenide quantum dots, lead selenide quantum dots, silver selenide quantum dots, and others (He et al., 2017; Sousa et al., 2020; Yuan et al., 2022), are severely restricted due to their toxicity, mainly caused by the presence of heavy metal ions. Recently, metal-free elemental selenium QDs (SeQDs) have emerged as a new type of fluorescent nanomaterial with the potential to replace traditional selenium-based QDs. This is due to their unique physicochemical properties, low cytotoxicity and good biocompatibility (Deng et al., 2023; Lv et al., 2023). Currently, there has been a dearth of literature discussing the properties and potential applications of SeQDs, despite their importance in biomedical applications. As such, there is a need to write a review paper to consolidate the research progress made in SeQDs. This mini review aims to provide an overview of the various approaches for synthesis of SeQDs, followed by discussions on their toxicity and biomedical applications, including bioimaging, biosensing, and disease therapy. The challenges and opportunities in SeQDs research, specifically in terms of synthesis and biomedical applications, are also discussed. Overall, this mini review hopes to shed light on SeQDs and encourage further research and practical applications by researchers.

## Synthesis

**FIGURE 1:**
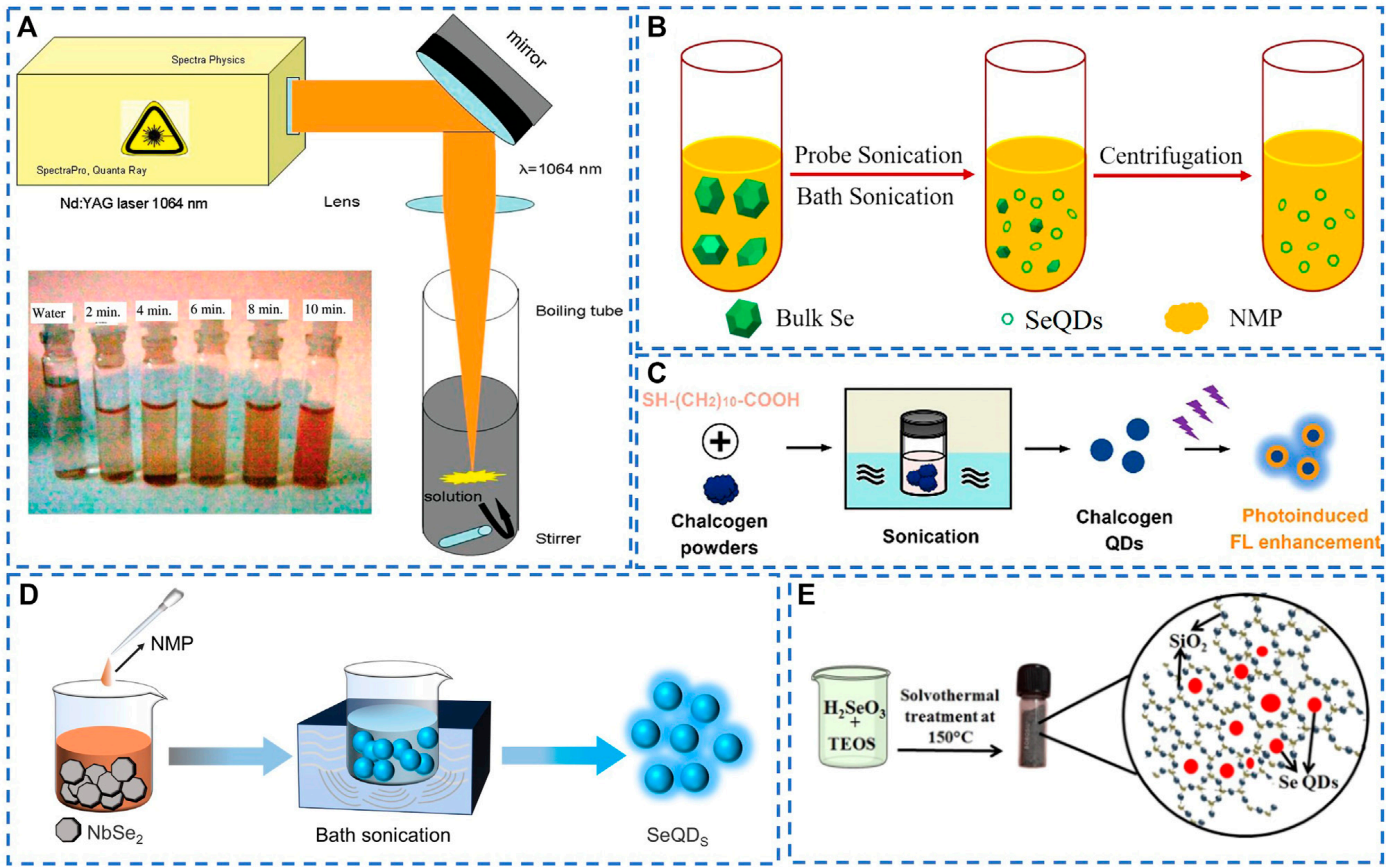
**(A)** Schematic diagram of preparation of SeQDs by laser irradiation. Reproduced, with permission, from Singh et al. (2010). Copyright 2010, American Chemical Society. **(B)** Ultrasonic assisted synthesis of SeQDs from bulk selenium powder. Reproduced, with permission, from Jiang et al. (2020). Copyright 2020, American Chemical Society. **(C)** Preparation of SeQDs by ultrasonic treatment of bulk selenium powder in the presence of thiolates. Reproduced, with permission, from Zhang et al. (2021). Copyright 2021, American Chemical Society. **(D)** Preparation of SeQDs by sonication treatment of NbSe_2_. **(E)** Solvothermal synthesis of SeQDs in the presence of TEOS. Reproduced, with permission, from Anupama et al. (2021). Copyright 2021, American Chemical Society.

The synthesis of SeQDs can be achieved through two strategies: the “top-down” and the “bottom-up” strategies, similar to other reported fluorescent QDs. The “top-down” strategy entails crushing the bulk selenium powder using physical forces, resulting in the production of SeQDs ranging in size from 2 to 10 nm. Singh et al. dispersed bulk selenium powder into a boiling tube filled with double steamed water and subjected it to irradiation using a pulsed Nd:YAG laser for 15 min. They successfully obtained SeQDs with a particle size of 2.74 nm (Singh et al., 2010). Similarly, Guisbiers et al. dispersed bulk selenium powder in ethanol and exposed it to laser beams at three different wavelengths, namely, 355, 532, and 1,064 nm. They discovered that the SeQDs obtained after 4–6 h of irradiation at any of the three wavelengths were all smaller than 4 nm (Guisbiers et al., 2015) (Figure 1A). Furthermore, they noted that the size and optical properties of the synthesized SeQDs were highly reliant on the duration of irradiation. Nevertheless, the efficiency of SeQDs preparation *via* laser irradiation is low, and it poses certain risks to human health. In addition to laser irradiation treatment, preparation of SeQDs through ultrasonication liquid-phase exfoliation of bulk selenium powder has also been reported. A study by Jiang et al. involved adding bulk selenium powder to N-methylpyrrolidone (NMP) solvent and subjecting the mixture to ultrasonic bath treatment at a temperature of 5 °C and ultrasonic power of 400 W (Jiang et al., 2020) (Figure 1B). This led to the successful synthesis of SeQDs with an average diameter of 4.9 nm, opening up new method for SeQDs synthesis. Another study by Zhang et al. also used bulk selenium powder in the presence of thiolates and NMP solvent to prepare SeQDs through ultrasonication treatment (Zhang et al., 2021) (Figure 1C). Initially, the 100 W power bath sonication was employed, followed by the application of 110 W tip sonication for the treatment process. They found that NMP was the most effective solvent among other solvents due to its surface tension matching with the surface energy of selenium powder. Furthermore, the SeQDs exhibited exceptional photostability, accompanied by a notable increase in fluorescence intensity with extended ultraviolet light irradiation time. This intriguing result presents a promising avenue for optimizing the optical properties of SeQDs.

Conversely, the “bottom-up” strategy involves the direct synthesis of SeQDs from selenium-based precursors by chemical reaction (Huang et al., 2020a). Thus far, two types of precursors have been utilized: elemental selenium powder and selenium-containing compounds. For instance, Yang and colleagues have successfully synthesized SeQDs by introducing selenium powder into a sodium sulfite solution along with bovine serum albumin. After adjusting the pH of the reaction mixture to 6, the mixture was incubated at varying temperatures and time intervals. It was discovered that amorphous SeQDs could be achieved by incubating the mixture at 20 °C for 12 h. On the other hand, crystalline SeQDs were obtained by incubating the mixture at 80 °C for 24 h. The average sizes of the amorphous and crystalline SeQDs acquired were reported to be 2.25 nm and 4.10 nm, respectively (Wang et al., 2016; Zhu et al., 2019). In addition, SeQDs with a mean size of 4.6 nm can be synthesized by introducing a mixture of selenium powder, potassium hydroxide, and hydrazine hydrate into a solution of κ-carrageenan (κ-CG) after heating at 70 °C under an argon atmosphere for 30 min (Lesnichaya et al., 2019). The authors proposed that hydrazine hydrate acts as a reducing agent, converting selenium powder into highly reactive selenide anions (Se^2-^) in an alkaline environment. It was observed that an aqueous solution of κ-CG can then oxidize the Se^2-^ anions to zero valent Se atoms.

Alternatively, the direct oxidation or reduction of selenium-containing compounds, such as H_2_SeO_3_ and NbSe_2_, to zero-valent Se atoms, has also been employed in the synthesis of SeQDs. For instance, Fujishima et al. demonstrated that UV irradiation of ethanol and methanol solutions containing H_2_SeO_3_ can yield highly dispersed SeQDs on the surface of TiO_2_. Interestingly, the average size of the SeQDs was found to increase as the irradiation time increased. Following a 2 h irradiation period, the average size of the SeQDs reached 8.7 nm (Fujishima et al., 2014). However, this method necessitates specific instrumentation and entails a complex operational procedure. Qian and colleagues utilized NbSe_2_, which is constrained with weak van der Waals forces, as a selenium precursor and added it to NMP. The mixture was then subjected to continuous high power ultrasonication of 500 W for 4 h. The resulting dispersion was then centrifuged, and the supernatant was collected to obtain SeQDs with an average size of 2.95 nm and a remarkable photoluminescence quantum yield of 22.7%, which is the highest reported quantum yield so far (Qian et al., 2017) (Figure 1D). Similarly, Guo and colleagues reported the synthesis of SeQDs by dissolving NbSe_2_ in distilled water and subjecting it to an autoclave reaction at 60 °C for 4 h. Vitamin C was subsequently added, and ultrasound treatment was performed at pH 8.0 for 3 h to yield SeQDs with an average size of approximately 5 nm. The prepared SeQDs exhibited favorable colloidal stability and maintain their size in pure water, PBS buffer (pH = 7.4), and cell culture medium. However, the time-consuming synthesis and intricate post-processing steps pose significant challenges for scalable production of SeQDs (Guo et al., 2021). To satisfy the need for solid-state fluorescent quantum dots (QDs) with anti-self-quenching properties, Anupama et al. utilized a solvation-assisted sol-gel approach to fabricate solid-state luminescent SeQDs with an average size between 3 and 8 nm (Anupama et al., 2021) (Figure 1E). Their findings suggest that the solvothermal decomposition of selenite leads to nucleation of triangular selenium nanocrystalline in the presence of tetraethyl orthosilicate (TEOS).

Various physical and chemical approaches have been developed for the synthesis of fluorescent SeQDs based on “top-down” and “bottom-up” strategies (Ruan and Zhou, 2022; Yang et al., 2023). Physical methods, such as laser irradiation and ultrasound, require advanced equipment and have high energy consumption but relatively low yield (Huang et al., 2023). Chemical methods, such as wet chemistry, offer higher yields but may involve the use of toxic ingredients, high temperatures, and high pressures, posing certain production risks (Yao et al., 2018; Gao et al., 2020). Therefore, there is a strong need for the development of a facile and effective approach to enable scalable synthesis of highly fluorescent SeQDs. On the other hand, biosynthesis technique is widely recognized as a clean, efficient, safe, and promising method for preparing nanoparticles. However, the biosynthesis of SeQDs has not been reported to date. It is anticipated that this technique will be employed in the future for the synthesis of fluorescent SeQDs.

## Biomedical applications

**FIGURE 2:**
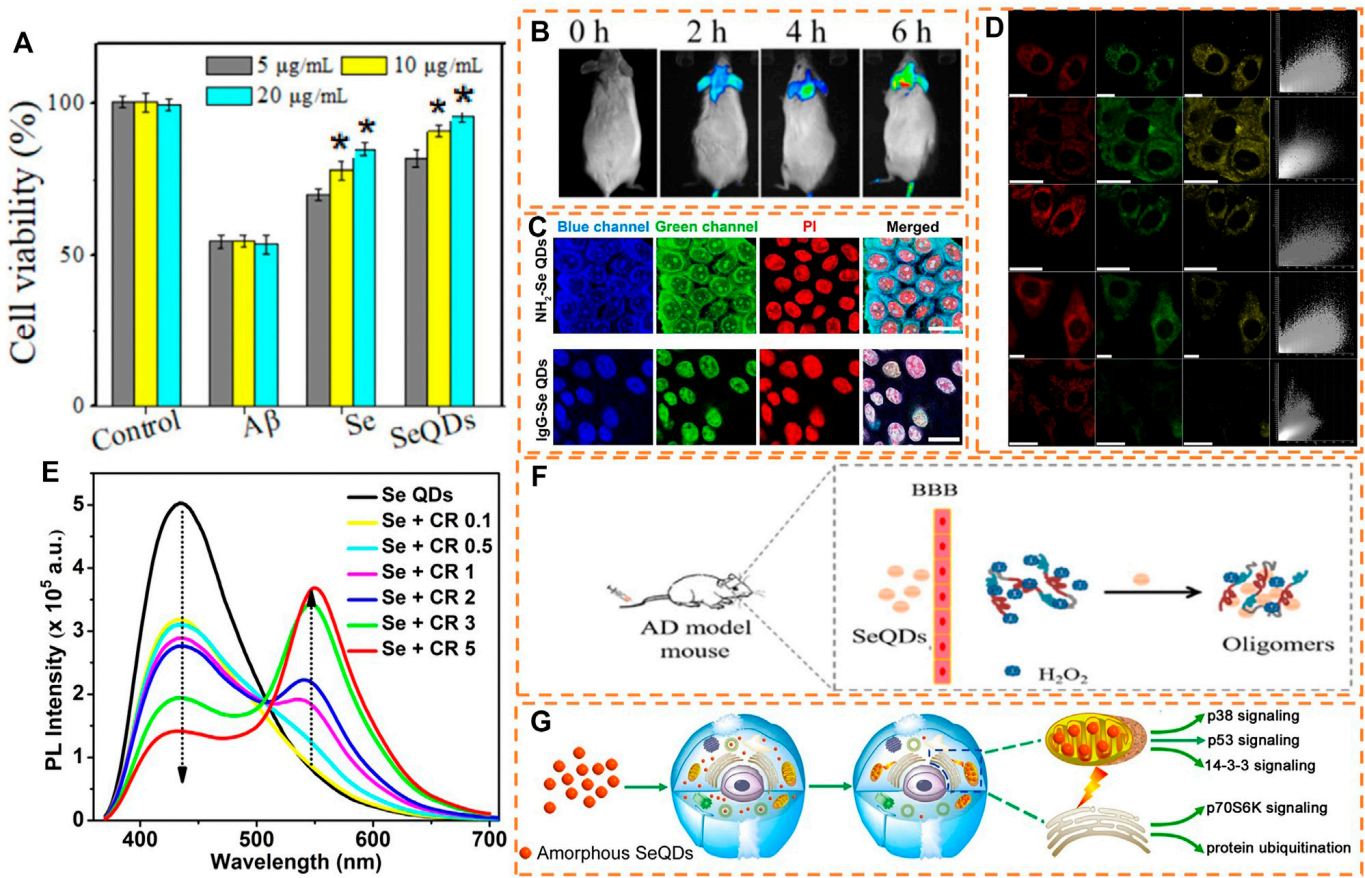
**(A)** Cytotoxicity assessment of SeQDs and **(B)** *in vivo* fluorescence imaging with SeQDs. Reproduced, with permission, from Guo et al. (2021). Copyright 2021, American Chemical Society. **(C)** LSCM fluorescence images of fixed/permeated cells treated with NH_2_-SeQDs and lgG-SeQDs. Reproduced, with permission, from Zhang et al. (2021). Copyright 2021, American Chemical Society. **(D)** LSCM fluorescence images of different cells incubated with SeQDs. Reproduced, with permission, from Wang et al. (2016). Copyright 2016, Springer Nature. **(E)** Fluorescence detection of curcumin by SeQDs. Reproduced, with permission, from Anupama et al. (2021). Copyright 2021, American Chemical Society. **(F)** Using SeQDs for dissociating Aβ fibrils and balancing ROS level to treat AD. Reproduced, with permission, from Guo et al. (2021). Copyright 2021, American Chemical Society. **(G)** Mechanism of anti-proliferation effect of SeQDs on cancer cells. Reproduced, with permission, from Wang et al. (2016). Copyright 2016, Springer Nature.

The increasing interest in the biomedical application of SeQDs has highlighted the importance of evaluating their potential toxicity. To assess SeQDs toxicity, researchers have broadly utilized cell viability tests with specific assays like MTT, CCK-8, and WST-1 (Kundu et al., 2019; Ahmadi et al., 2022). For example, in a study by Guo et al., MTT assays were employed to evaluate the cytotoxicity of SeQDs on SH-SY5Y cells, demonstrating that SeQDs had much lower cytotoxicity than elemental selenium powder (Guo et al., 2021) (Figure 2A). Likewise, Zhang and colleagues examined the toxic effects of SeQDs on HeLa and HEK-293 cells and found that even after incubation with 1 mg/mL SeQDs for 24 h, the cell viability remained at 80% (Zhang et al., 2021). The results of these studies provide a solid foundation for further research on the biomedical applications of SeQDs, as their low cytotoxicity suggests a promising safety profile.

Currently, SeQDs find wide application in the field of biomedicine, particularly in bioimaging, biosensing, and diagnosing treatment. Bioimaging plays a critical role in enhancing our comprehension of cellular structures and physiological processes in organisms. Among the diverse range of fluorescent nanomaterials, SeQDs stand out due to their unique photoluminescence properties. What sets SeQDs apart is the fact that selenium is a necessary trace element in the body (Lian et al., 2019; Zhou et al., 2022a), enhancing their significance in bioimaging. Guo et al. conducted a comprehensive study by injecting SeQDs into mice, which resulted in the observation of the fluorescent signal of SeQDs entering the brain 2 h later (Guo et al., 2021) (Figure 2B). With the progress of time, the fluorescence signal in the brain gradually intensified, eventually reaching its highest value at 6 h. Notably, the organs of the SeQDs-treated mice demonstrated an intact structure, without any pathological changes or damage to organs when compared to the control group. These results provide evidence for the exceptional biosafety of SeQDs *in vivo*, as well as establish their effectiveness as a fluorescent probe for bioimaging. Zhang et al. synthesized IgG tailored SeQDs (IgG-SeQDs) and utilized them in immunofluorescence imaging (Figure 2C) (Zhang et al., 2021). The laser scanning confocal microscope (LSCM) images clearly demonstrated the successful binding of IgG to SeQDs and their excellent co-localization with the nucleo-stained propyl iodide (PI), confirming the exceptional nuclear staining ability of IgG-SeQDs. In a separate study, Wang et al. employed SeQDs as a fluorescent probe for imaging HeLa cells. Figure 2D illustrates the intense blue and green fluorescence observed in the HeLa cells after incubation with SeQDs, upon excitation by 405 nm and 488 nm laser irradiation, respectively (Wang et al., 2016). However, most reported SeQDs can only emit blue and green fluorescence, limiting their potential for bioimaging. Additionally, the spontaneous fluorescence of cells and biological tissues may interfere with the fluorescence of SeQDs. Therefore, it is necessary to develop fluorescent SeQDs with long wavelength emissions, such as red fluorescence and near-infrared fluorescence.

The unique optical properties and surface characteristics of SeQDs make them a valuable fluorescent nanomaterial for biosensing applications (Huang et al., 2021). For example, Anupama et al. demonstrated the use of SeQDs as a fluorescent probe for sensing curcumin (CR) (Figure 2E) (Anupama et al., 2021). The addition of CR led to a significant decrease in the fluorescence intensity of SeQDs, which was attributed to the presence of the internal filtration effect (IFE). This effect occurs when the absorption spectrum of CR overlaps well with the excitation and emission spectra of SeQDs. Additionally, selenium is an essential co-contributor to the optimal function of the antioxidant enzyme glutathione peroxidase, thereby playing a crucial role as a redox regulator in maintaining cellular homeostasis. Consequently, SeQDs hold great potential for anticancer activity and pro-oxidation properties in the treatment of various diseases (Liu et al., 2020; Zhou et al., 2022a; Zhou et al., 2022b). The unique attributes of SeQDs enable them to be utilized both as a fluorescent probe and a therapeutic agent in practical applications. For instance, Guo et al. have explored the potential of SeQDs as a valuable tool for detecting and monitoring Alzheimer’s disease (AD) (Guo et al., 2021). By employing fluorescence tracking technology, the researchers discovered that SeQDs entered the brain within approximately 2 h of their injection in mice. The fluorescence signals in the brain steadily increased over time and peaked after 6 h, indicating that SeQDs can efficiently traverse the blood-brain barrier (BBB) and steadily accumulate in the brain (Figure 2F). Aside from its ability to inhibit amyloid-beta (Aβ) aggregation, which is the culprit behind AD, SeQDs can also reduce Aβ-mediated cytotoxicity, thus blocking the progression of AD. This, in turn, helps minimize oxidative stress, restore mitochondrial function, maintain nerve cell stability and safeguard nerve cells against oxidative stress. In addition, the researchers observed higher levels of fluorescence signals in the livers and kidneys of mice that were injected with SeQDs, indicating that most of the selenium is rapidly metabolized after entering the body. Additionally, H&E staining of the heart, liver, spleen, lung, and kidney of mice revealed that compared to the control group, the organs of the SeQDs-treated mice were structurally intact, and no pathological changes were evident. This finding suggests that SeQDs are unlikely to cause any damage to various tissues and organs. Hence, SeQDs have an edge over conventional single-target drugs in the treatment of AD, providing a fresh avenue towards the prevention and treatment of neurodegenerative diseases. The anti-cancer activity of Se-containing nanoparticles may be strongly influenced by their surface and crystalline characteristics. As reported by Chen’s group, the use of L-glutathione modified Se nanoparticles leads to a higher reduction in reactive oxygen species and mitochondrial breakage compared to D-glutathione modified ones. This, in turn, prevents the oxidative damage of INS-1 cells caused by palmitic acid. (Huang et al., 2020b). Similarly, Wang and colleagues observed that amorphous-SeQDs exert their anti-cancer effects primarily through their uptake and localization in mitochondria. This leads to severe damage to mitochondrial membranes, depletion of mitochondrial potential, induction of apoptosis, and cell cycle arrest in the S phase, which ultimately hinder the growth and proliferation of tumor cells. Conversely, crystalline-SeQDs were found to have a weaker impact (Wang et al., 2016). Furthermore, these effects were attributed to the unique ability of SeQDs to differentially regulate 61 proteins and several signaling pathways related to stress response, protein synthesis, cell migration, and cell cycle (Figure 2G). These findings provide a deeper understanding of the mechanisms driving the anti-proliferative effects of nanoparticles on cancer cells, and suggest that SeQDs may represent a promising nanomaterial for cancer treatment. Atherosclerosis is a condition characterized by the accumulation of a lipid layer in the arterial wall, reducing artery elasticity and narrowing the arterial lumen (Dong et al., 2020). Endothelial dysfunction is a leading cause of atherosclerotic plaque and a risk factor for myocardial infarction rupture (Zhao et al., 2023). Zhu and colleagues found that SeQDs can inhibit the activity of Na+/H+ exchanger 1 (NHE1) and impair calcium ion/calpain signaling, effectively improving endothelial cell relaxation, preventing endothelial dysfunction, and limiting the growth of atherosclerotic plaques (Zhu et al., 2019). Overall, SeQDs have demonstrated remarkable potential in the treatment of various diseases, including cancer, Alzheimer’s disease, and atherosclerosis, resulting in increased interest and attention from researchers in this field.

## Conclusion and perspectives

Conventional semiconductor QDs that contain selenium element, such as CdSe QDs, Ag_2_Se QDs, and PbSe QDs, suffer from high toxicity. Consequently, their application in biomedical fields remains limited. However, SeQDs, being a novel form of fluorescent nanomaterial, possess several advantages such as low toxicity, small particle size, and unique optical and surface properties. Moreover, selenium is a requisite trace element in the human body, which makes SeQDs more preferable as fluorescent QDs in *in-vivo* research, potentially avoiding rejection between the nanomaterial and organisms. Therefore, SeQDs possess substantial potential in biomedical research, especially in biological imaging, sensing, diagnosis, and treatment.

While researchers have made remarkable progress in their studies, SeQDs still possess certain limitations. Notably, their photostability diminishes significantly when exposed to excitation light for prolonged periods, leading to a decline in fluorescence. Although several methods have been developed to improve their photostability, the task of generating stable SeQDs on a larger scale to meet the growing demand remains challenging. Furthermore, achieving scalable synthesis of highly fluorescent SeQDs is yet another hurdle. Existing approaches reported so far can only produce minimal quantities of SeQDs, falling short of practical application requirements. When considering bioimaging applications, it is important to note that SeQDs generally emit in the blue-green light range. Unfortunately, this range of light can be absorbed by organisms, and the fluorescence from the organism may impact imaging accuracy. Therefore, exploring new SeQDs that emit in the red or near-infrared range is imperative for more precise bioimaging. Additionally, SeQDs can be paired with specific targeted molecules to produce efficient and sensitive bioprobes that allow for the monitoring of molecular-level reactions in organisms through advanced optical imaging technology. This aspect is also a vital direction for SeQDs research. While SeQDs have proven to be effective biosensors for various substances such as metal ions and drug molecules, their application in this field remains limited. Further research in this area could lead to potentially groundbreaking discoveries. Furthermore, SeQDs have shown promising advantages in the realms of diagnosis and treatment, but clinical trials have yet to be conducted, presenting a pressing issue that requires urgent attention. Additionally, SeQDs may find utility in agriculture by improving the cultivation of plants and vegetables, resulting in an abundance of produce that is rich in selenium. As such, using SeQDs as a selenium supplement to prevent various diseases is also an emerging trend in the field. In addition, SeQDs possess unique properties that make them promising for use in active food packaging. Their exceptional antibacterial and antioxidant properties offer a potential solution to enhance the functionality of conventional packaging by providing extended food shelf life during transportation and storage while preserving food quality.
